# Supplementary material for: Initial Steps towards Spatiotemporal Signaling through Biomaterials Using Click-to-Release Chemistry
Source: Pharmaceutics. 2022 Sep 21;14(10):1991. doi: 10.3390/pharmaceutics14101991 (PMC9610979; doi:10.3390/pharmaceutics14101991)
Supplement: Supplementary file 1 [file pharmaceutics-14-01991-s001.zip › pharmaceutics-1918284-supplementary.pdf]

Supplementary data file

Gansevoort M, Merx J, Versteeg EMM, Vuckovic I, Boltje TJ, van Kuppevelt TH and Daamen WF

Initial steps towards spatiotemporal signaling through biomaterials using click-to-release chemistry

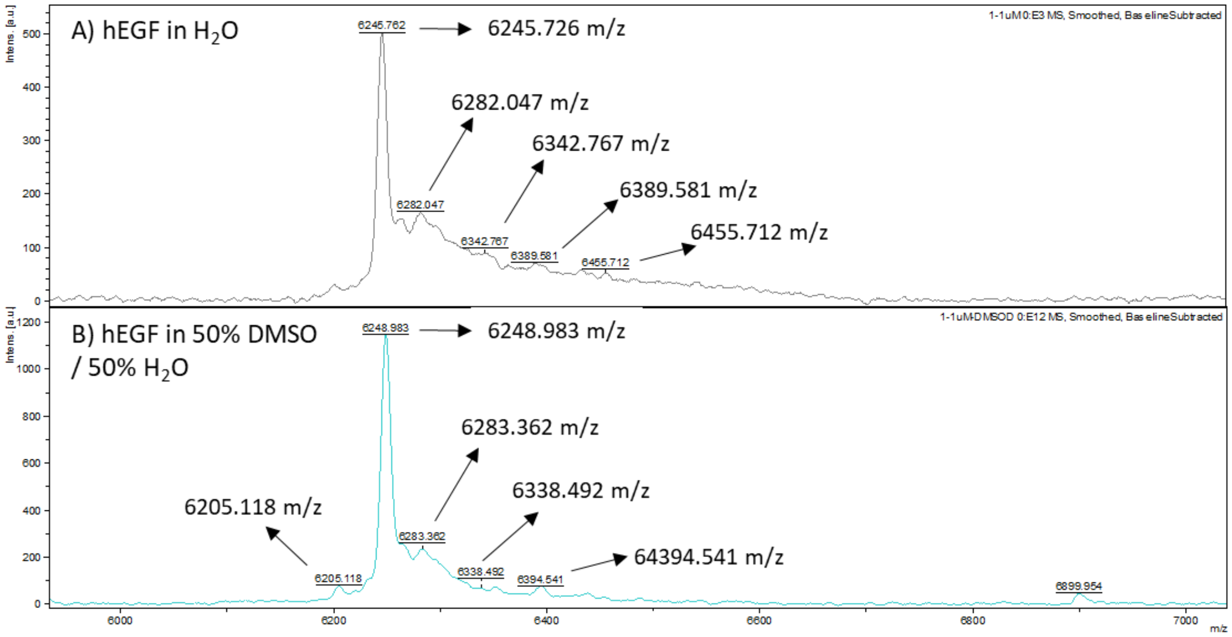

Figure S1 Additional mass spectra of hEGF. Mass spectra of hEGF in A) 100% H_2O and B) 50% DMSO/50% H_2O indicate no effect of the presence of DMSO in the sample buffer of hEGF.

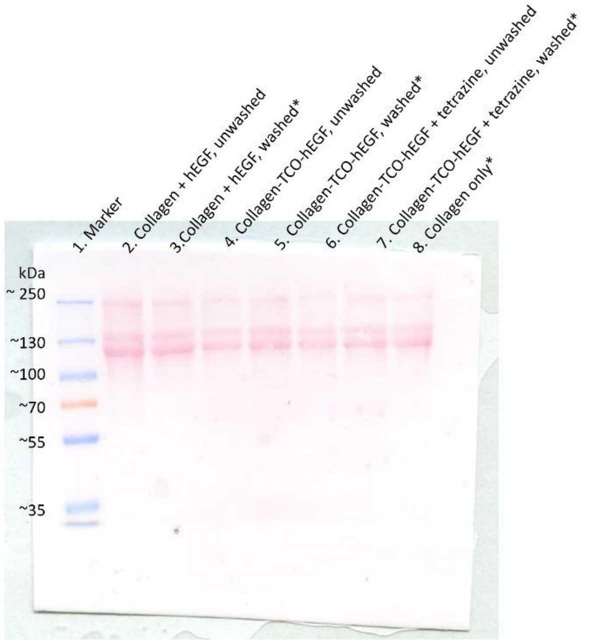

Original image of blot of main text Figure 3D, where total protein is stained with Ponceau S and protein marker in lane 1 is prestained in blue and orange. Collagen scaffolds were combined with sample buffer, denatured at 100°C for 10 min, centrifuged and loaded on gel. In unwashed collagen scaffolds (lane 2, 4, 6) unbound components were not washed away, whereas in washed scaffolds (lane 3, 5, 7) aspecifically bound components were removed by washings. Lanes marked with * were used for the main text figure.

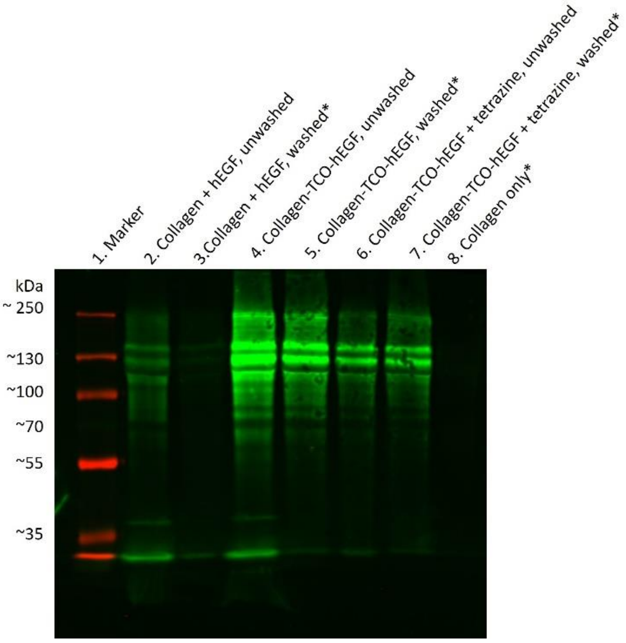

Original image of Western blot of main text Figure 3E, where the protein marker (lane 1) is visible in red and hEGF is labeled in green. Collagen scaffolds were combined with sample buffer, denatured at 100°C for 10 min, centrifuged and loaded on gel. In unwashed collagen scaffolds (lane 2, 4, 6) unbound components were not washed away, whereas in washed scaffolds (lane 3, 5, 7) aspecifically bound components were removed by washings. Lanes marked with * were used for the main text figure.

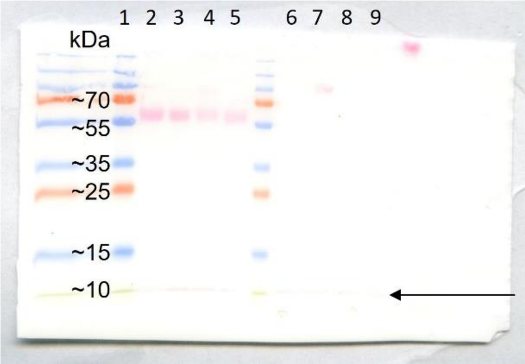

Original image of blot of main text Figure 4A, where total protein is stained with Ponceau S and protein marker in lane 1 is prestained in blue and orange. Lanes 2 and 3 were used in the main text figure. Lanes 4 and 5 contain samples made with a reversed reaction order: TCO was first conjugated to BSA and the TCO-BSA was ligated to hEGF. Lanes 6, 7, 8 and 9 contain samples of hEGF with different protein denaturation methods.

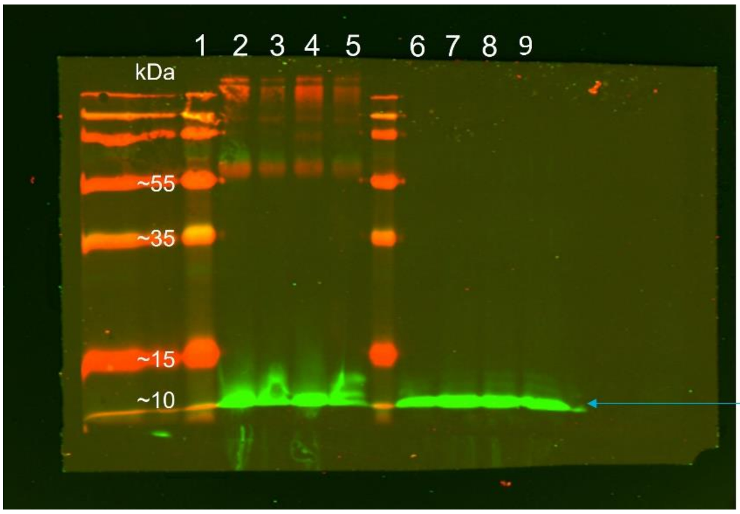

Original Western blot of main text Figure 4B, where the protein marker (lane 1) is visible in red, BSA is labeled in red and hEGF in green. Lanes 2 and 3 were used in the main text figure. Lanes 4 and 5 contain samples made with a reversed reaction order: TCO was first conjugated to BSA and the TCO-BSA was ligated to hEGF. Lanes 6, 7, 8 and 9 contain samples of hEGF with different protein denaturation methods.

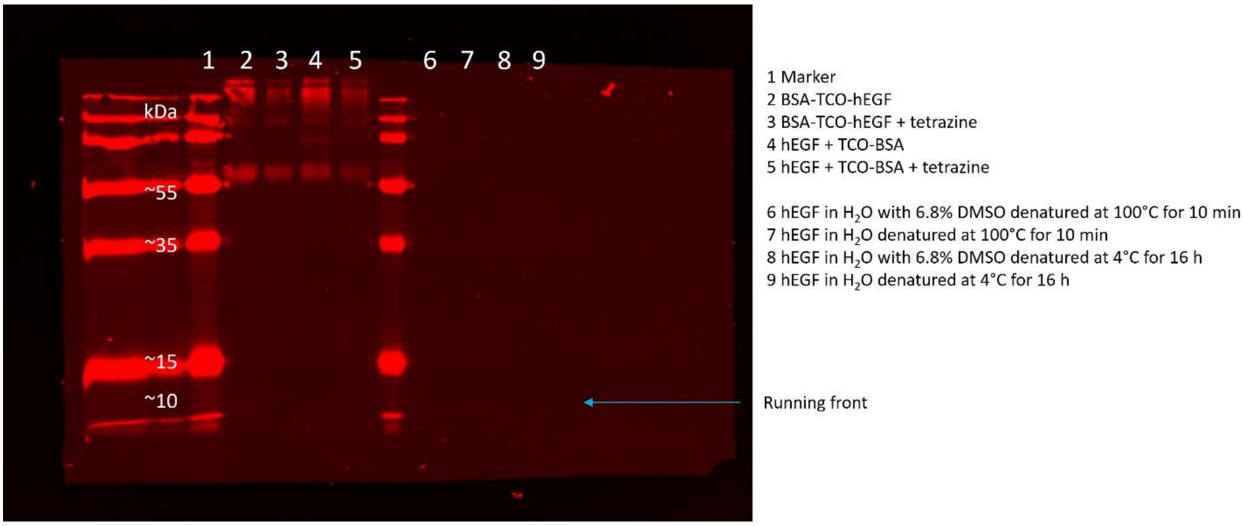

Original image of Western blot of main text Figure 4C, where BSA in lanes 2-5 is immunolabeled in red. Lane 1 contains protein marker, lanes 2 and 3 were used in the main text figure.

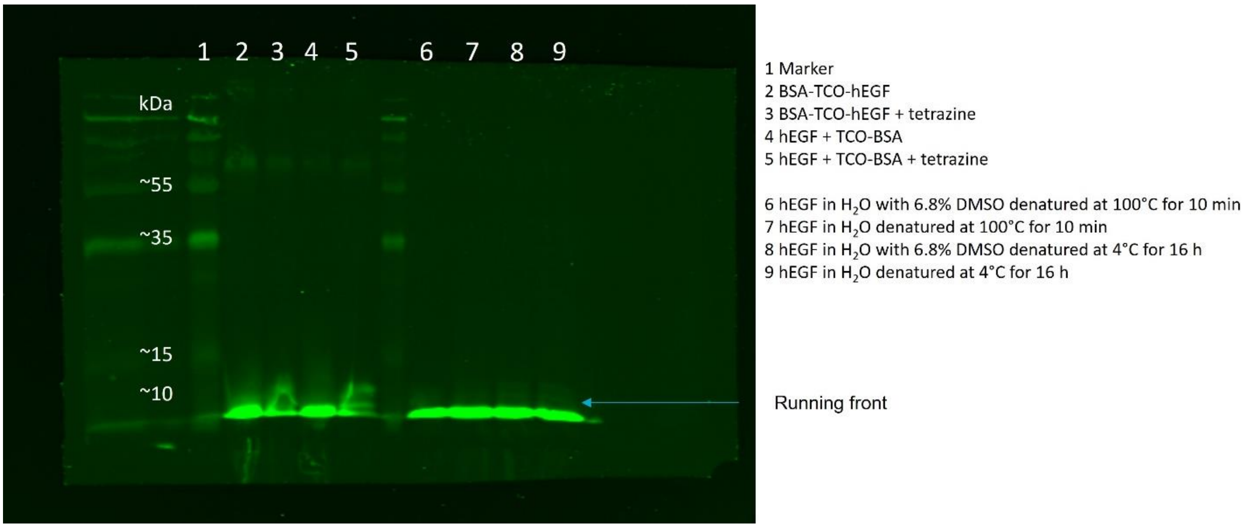

Original image of Western blot of main text Figure 4D, where hEGF in lanes 2-9 is immunolabeled in green. Lane 1 contains protein marker, lanes 2 and 3 were used in the main text figure.
